# Experimental Investigation of Flexural Behavior of Ultra-High-Performance Concrete with Coarse Aggregate-Filled Steel Tubes

**DOI:** 10.3390/ma14216354

**Published:** 2021-10-24

**Authors:** Fanghong Wu, Yanqin Zeng, Ben Li, Xuetao Lyu

**Affiliations:** 1 Advanced and Sustainable Infrastructure Materials Group, School of Transportation, Civil Engineering & Architecture, Foshan University, Foshan 528000, China; lxtwww30@fosu.edu.cn; 2 School of Civil Engineering, Wuhan University, Wuhan 430072, China; yanqinzeng@whu.edu.cn

**Keywords:** UHPC with coarse aggregate (CA-UHPC), concrete-filled steel tube, circular section, flexural stiffness, flexural capacity

## Abstract

This paper presents an experimental investigation of flexural behavior of circular ultra-high-performance concrete with coarse aggregate (CA-UHPC)-filled steel tubes (CA-UHPCFSTs). A total of seven flexural members were tested under a four-point bending load. The failure modes, overall deflection curves, moment-versus-curvature relationships, moment-versus-strain curves, strain distribution curves, ductility, flexural stiffness and ultimate flexural capacity were evaluated. The results indicate that the CA-UHPCFSTs under bending behaved in a good ductile manner. The CA-UHPC strength has a limited effect on the ultimate flexural capacity, while the addition of steel fiber can improve the ultimate flexural capacity. Increasing the steel tube thickness leads to higher flexural stiffness and ultimate flexural capacity. There is a significant confinement effect between the steel tube and the CA-UHPC core in the compressive zone and centroidal plane after the specimen enters the elastic-plastic stage, while the confinement effect in the tensile zone is minimal. Moreover, the measured flexural stiffness and ultimate flexural capacity were compared with the predictions using various design specifications. Two empirical formulas for calculating the initial and serviceability-level flexural stiffness of CA-UHPCFSTs are developed. Further research is required to propose the accurate design formula for the ultimate flexural capacity of CA-UHPCFSTs.

## 1. Introduction

Concrete-filled steel tubes (CFSTs) combine the advantages and overcome the shortcomings of concrete and steel materials [1]. Due to the confinement action of the steel tube, the compressive strength and ductility of concrete core can be substantially improved. Conversely, the infilling of concrete effectively delays the global and local buckling of the steel tube. Furthermore, the limitations of natural resources and the seriousness of environmental disruption have produced an urgent need for sustainable and high-performance materials. Ultra-high-performance concrete with coarse aggregate (CA-UHPC), an innovative cement-based composite material with ultra-high strength, good ductility, superior durability and relatively low cost, has broad application prospects in bridges, roads, ocean engineering, military buildings and infrastructure [2].

The properties of UHPC have been extensively investigated in the last 30 years. The existing studies indicate that UHPC without the employment of steel fibers exhibits highly brittle features in compression, and post-peak brittleness limits its application [3,4]. The incorporation of steel fibers can improve the ductility of UHPC, but it has a slight influence on the compressive strength and elastic modulus [5,6,7], while also increasing the preparation cost of UHPC. Wang et al. [8] and Hoang et al. [9] reported that UHPC-filled steel tubes (UHPCFSTs) are an effective way of further enhancing the compressive strength and ductility of UHPC, due to the confinement effect of the steel tube. This combination of UHPC and steel tube can enhance the ultimate carrying capacity of the member while reducing the deadweight of the structure, thus meeting the requirements of super-large, ultra-high and large-span constructions. Over the past decade, the mechanical behavior of UHPCFST columns under axial compression has been investigated [10,11,12,13]. Chen et al. [14] carried out an experimental study on the structural behavior of UHPCFST members, with the test results demonstrating that the ductility of UHPCFST columns was much better than that of the ordinary CFSTs, and that the existing design codes were not accurate for the prediction of ultimate strength; thus, a simplified calculation method for the predicting of ultimate strength was developed. Yan et al. [15] performed an investigation of 32 short square UHPCFST columns subjected to axial compression. It was found that the UHPCFST columns exhibited a good ductile behavior, and the effect of the confinement index on ductility was more pronounced than that of the ultimate strength. However, the conclusions reported Xu et al. [16] and Wei et al. [17] indicate that the confinement enhancement effect on UHPC infill provided by the steel tube is weaker than that on normal-strength concrete or high-strength concrete. Normally, the confinement effect provided by the steel tube on UHPC infill is produced when the load reaches nearly 80~90% of the ultimate carrying capacity [8], lagging behind that of the normal CFST column, which corresponds to about 75% of the ultimate carrying capacity. It can be inferred that there are substantial differences in the mechanical properties and the interaction between steel tube and concrete core for ordinary CFST and UHPCFST columns.

CFSTs are usually subjected to axial compression and bending moment simultaneously, especially during earthquakes, and are barely ever used as pure bending members in practical projects. However, despite the pure bending state being a special case for column-beams, the study of the flexural behavior of CFST can help in further understanding the mechanical behavior of CFST column-beams under complex loading states, in which the flexural capacity is a critical reference point in the compression–moment interaction diagram. To date, several investigations have been implemented to study the flexural behavior of ultra-high-strength concrete or UHPC-filled steel tube beams. For example, Xiong et al. [18] experimentally investigated the flexural behavior of CFSTs with high-tensile steel and ultra-high-strength concrete. It was found that EC 4 [19] underestimated the plastic moment resistance and overestimated the effective section flexural stiffness. Guler et al. [20] conducted an experimental investigation of the flexural behavior of square UHPCFST, the results demonstrated that the predictions of ultimate flexural capacity from EC 4 were conservative. Li et al. [21] conducted an experimental investigation of the flexural performance of UHPC-filled high-strength steel tube (UHPCFST) and double-skin high-strength steel tube (UHPCFDST), and an analytical model was developed to predict the ultimate flexural capacity of UHPCFST and UHPCFDST members.

As mentioned above, the current research on UHPCFST members is mainly focused on compressive behavior. Moreover, the flexural performance of UHPC materials has also been widely studied, with the flexural strength and ductility of UHPC being superior to those of normal and high-strength concrete, as well as to those of fiber-reinforced concrete [3,4,22,23]. However, there is a lack of work investigating CFST beams infilled with ultra-high-performance concrete under pure bending, especially for UHPC with coarse aggregate-filled steel tubes (CA-UHPCFSTs). The mechanical properties of CA-UHPC are different from those of UHPC, because the moderate addition of coarse aggregate can increase the compressive strength, while decreasing the flexural strength [6]. Furthermore, various design codes and specifications can be employed to predict the flexural strength and flexural stiffness of normal-strength concrete-filled steel tubes, including the European code EC 4 [19], the American specification AISC/LRFD [24], the Japanese standard AIJ [25], the Australian standard AS 5100.6 [26], the British standard BS 5400-5 [27] and the Chinese code GB 50936 [28]. However, most of these design codes or specifications limit the strength of concrete and steel. For example, AISC/LRFD limits the compressive strength of normal-strength concrete to within the range of 21~70 MPa, while GB 50936 is applicable to concrete with a compressive strength in the range of 30~80 MPa. Meanwhile, the compressive strength of UHPC is normally greater than 120 MPa. Therefore, it is vital to intensively explore the flexural behavior of CA-UHPCFST members under pure bending load in order to better understand the mechanical performance of CA-UHPCFST column-beams under complex loading states, and to assess the feasibility of the current design codes or specifications for predicting the flexural strength and flexural stiffness of CA-UHPCFSTs.

This paper presents an experimental investigation of seven circular CA-UHPCFST beams subjected to four-point bending load. The considered parameters include steel tube thickness, CA-UHPC strength and the addition of steel fiber. The failure modes, moment–curvature relationships, moment-versus-strain curves, strain distribution curves, ductility, composite action, flexural stiffness and ultimate flexural capacity are presented. Additionally, the comparisons between the test results and the predictions from the current design codes or specifications were performed to evaluate the feasibility for predicting the flexural stiffness and ultimate flexural capacity of CA-UHPCFST member under bending.

The structure of this paper is as follows: in Section 2, the test design, materials properties, test setup and the loading procedure are presented. In Section 3, the test results, including failure modes, moment–curvature relationships, moment-versus-strain curves, strain distribution curves, ductility, composite action, flexural stiffness and ultimate flexural capacity, are presented and discussed. In Section 4, the experimental values of flexural stiffness and ultimate flexural capacity are compared with the predictions of design codes, and the feasibility of codes used for CA-UHPCFST members under bending are evaluated. In Section 5, the main conclusions of this paper are presented.

## 2. Experimental Program

### 2.1. Test Design

Seven CA-UHPCFST beams were classified into two groups on the basis of steel tube thickness and CA-UHPC type. All specimens had a same length of 1400 mm, and the effective length between the two supports, *L*_e_, was fixed at 1200 mm. The tubes were fabricated using seamless steel tube with the same external diameter of 114 mm, but different nominal wall thicknesses (*t*) of 4.5 mm, 6 mm, 8 mm and 10 mm were considered. Two steel plates with dimensions of 140 mm × 140 mm × 10 mm were welded at the both ends to avoid the relative slip between the steel tube and CA-UHPC infill during the loading process. The nomenclature of the specimen is as follows: BC32S6, where ‘B’ represents the bending specimen, ‘C32’ is defined as the type of CA-UHPC, with the mixture containing 30% coarse aggregate and 2% steel fiber by the volume fraction, and ‘S6’ indicates that the nominal thickness of steel tube is 6 mm. The details of all specimens used in the present study are summarized in Table 1.

### 2.2. Materials Properties

#### 2.2.1. Steel

**Figure 1:**
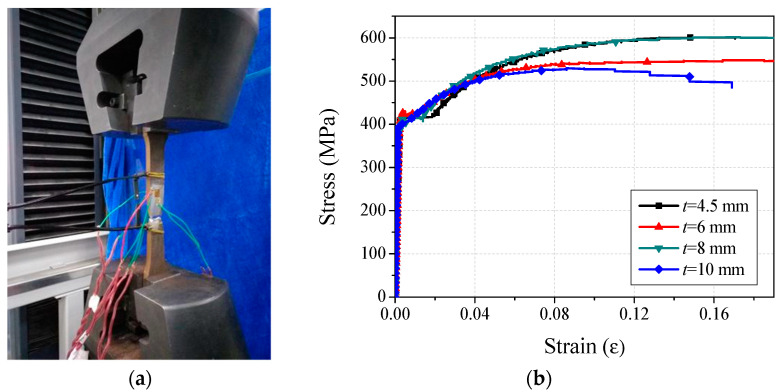
Test setup and stress-strain curves of steel tensile coupons. (**a**) Tensile coupon test setup; (**b**) stress-strain curves of steel tensile coupons.

All specimens were fabricated by the same seamless steel tube with a nominal yield strength of 390 MPa. The mechanical properties of steel tube were determined from the tensile coupon tests. Three identical tensile coupons for each thickness steel tube, cut from the seamless steel tube in the longitudinal direction, were prepared and tested according to Chinese codes of Metallic Materials GB/T 228.1-2010 [29]. A calibrated extensometer (Sanjing, Changchun, China) with a gauge length of 50 mm was fixed at the middle of the coupon to measure the longitudinal deformation, and two pairs of strain gauges were attached in the center to measure the longitudinal and transverse strains, as shown in Figure 1a. The typical stress-strain curves of the tensile coupons for different wall thicknesses of steel tubes are plotted in Figure 1b. The material properties of the steel tube obtained from the tensile coupon test, including the average values of yield strength (*f*_y_), ultimate tensile strength (*f*_u_) and elastic modulus (*E*_s_), are summarized in Table 2.

#### 2.2.2. UHPC with Coarse Aggregate

**Figure 2:**
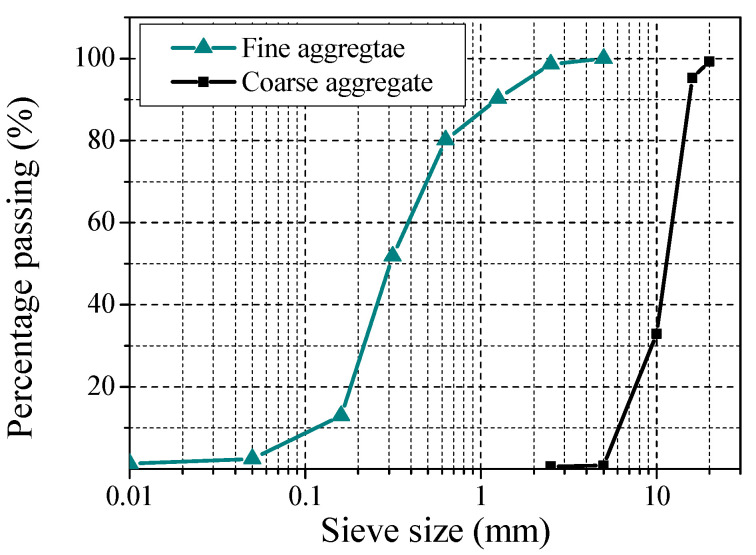
Grading curves of fine and coarse aggregates.

Four CA-UHPC mix proportions were designed, as listed in Table 3, where ‘C32’ represents the mixture incorporating 30% coarse aggregate and 2% steel fiber by volume fraction, ‘C20’ denotes that about 15% coarse aggregate by volume fraction was added to the mixture and no steel fiber was incorporated. The chemical composition of cement, silica fume and fly ash were determined via X-ray fluorescence (XRF, Bruker AXS, Karlsruhe, Germany), as listed in Table 4. Straight copper-coated steel fiber with a diameter of 0.6 mm and a length of 13 mm was added into the mix, the yield strength was greater than 2200 MPa. Polypropylene fiber (PPF) with a length of 19 mm and an aspect ratio of 396, density of 0.91 g/cm^3^, and tensile strength of 400 MPa was adopted in the mixture. River sand with an apparent density of 2650 kg/m^3^ and grain size below 2 mm was applied. Polycarboxylate superplasticizer (SP) with a water-reducing ratio of 30% and a solid content of 40% was applied to reduce the water consumption and improve the flowability of CA-UHPC. Crushed granite with particle size ranging from 5 mm to 20 mm was used, and the apparent density is about 2725 kg/m^3^. The grading curves of fine and coarse aggregates are shown in Figure 2.

The raw materials of CA-UHPC were mixed in a vertical axis planetary stirrer (Co-Nele, Qingdao, China) using the following procedures: firstly, the coarse aggregate and river sand were mixed 3 min; secondly, the cement, silica fume and fly ash were poured into the mixture to mix another 3 min; thirdly, about 75% of the superplasticizer was dissolved in the water and the polypropylene fiber was dispersed evenly in the solution, and then poured into the mixture for mixing 3~5 min; finally, the steel fibers were uniformly distributed in the paste, and the remaining water-reducer was added until good fluidity was achieved. The fresh CA-UHPC was poured into the hollow steel tube in four layers, and each layer was compacted by an internal vibrator. After the last layer was vibrated, an additional layer paste was flattened on the top surface until no bubbles overflowed. The top of the specimen was covered by a plastic sheet to prevent moisture evaporation and avoid separation between the steel tube and the concrete core due to the shrinkage of the CA-UHPC infill. Before the test, the other steel plate was welded on the top of the specimen. All CA-UHPCFST specimens were cured at ambient temperature and tested after 28 days

To determine the mechanical properties of the CA-UHPC infill, three cubes with sides of 100 mm and three prisms with dimensions of 100 mm × 100 mm × 300 mm were prepared along with the casting of the beams. Specifically, the prisms were sealed with tinfoil to simulate the real curing condition of CA-UHPC infill in CA-UHPCFST specimens. The cubes and prisms were cured under the same conditions as the beams and tested on the same day as the members. The material properties of each batch of CA-UHPC are summarized in Table 3.

### 2.3. Experimental Setup and Loading Procedure

**Figure 3:**
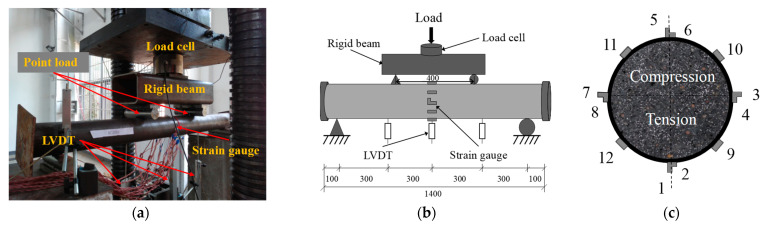
Experimental setup and locations of strain gauges. (**a**) Experimental setup; (**b**) schematic diagram (Unit: mm); (**c**) strain gauge locations.

The bending tests were conducted using a Universal Testing Machine (UTM, Hongshan, Tianshui, China) with a capacity of 5000 kN. All specimens were simply supported by two metal rollers on a loading frame, allowing the specimen to rotate about the bending axis and move along the in-plane longitudinal direction. A pure bending load with a constant moment region of 400 mm was applied via a rigid cross beam installed at one-third of the middle span, as shown in Figure 3. The applied axial load was collected by a 500 kN load cell. Three linear variable differential transducers (LVDTs, Zhongce, Yangzhou, China) were symmetrically installed to measure the in-plane vertical deflection profile. Four groups of strain gauges, consisting of a pair of mutually perpendicular gauges, were pasted symmetrically at the mid-span section to measure the longitudinal and transverse strains. Another four strain gauges were attached longitudinally to measure the longitudinal strain at 1/4 and 3/4 height of the mid-span section, as shown in Figure 3c. A photograph of the experimental setup and the locations of the strain gauge arrangements are presented in Figure 3. The data of the load cell, LVDTs and strain gauges were collected at a constant frequency of 2 Hz using an automatic acquisition system DH 3816N.

The loading process was divided into two stages: preloading and formal loading. The specimen was firstly preloaded to 20% of the predicted flexural capacity in order to guarantee good contact between the specimen and the facility. Step loading was performed during the formal loading. The load was firstly applied at increments of 10% of the predicted ultimate capacity before the yielding of the specimen, and each loading interval was maintained for 2~3 min. After the yielding of the specimen, displacement control was implemented at a rate of 1 mm/min until the end of the test. The test was terminated when the mid-span deflection exceeded 80 cm (*L*_e_/15).

## 3. Test Results and Discussion

### 3.1. Failure Modes

**Figure 4:**
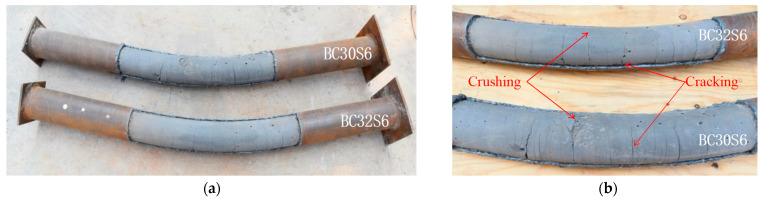
Failure modes of specimen and CA-UHPC infill. (**a**) Specimen; (**b**) CA-UHPC infill.

All specimens behaved in a good ductile manner throughout the entire loading process; when the mid-span maximum deflection reached 80 cm, the flexural capacity still kept increasing linearly. In the elastic stage, no visible flection could be observed, and the surface of the steel tube also showed no obvious change. In the elastic–plastic stage, the mid-span deflection gradually became obvious, and the strain in the tensile flange at the mid-span section exceeded the yield strain of the steel, with some rust falling off the steel tube. As the load continued, the specimen made a noise during the first two stages, indicating the cracking of the CA-UHPC core in the tensile zone. In the plastic hardening stage, the moment linearly increased at a slower rate, but the deflection increased dramatically. The typical failure modes of the CA-UHPCFST specimens under bending are illustrated in Figure 4. It can be seen that the specimens exhibited global buckling along the length. A few wrinkles can be seen on the compression flange for the thin-thickness specimen at the mid-span section; no tensile fracture on the tensile flange of steel tube was observed before the termination of the test. Similar failure modes of UHPCFST beams under bending were observed by Guler et al. [20], Li et al. [21] and Lu et al. [30], who found that CFST beams infilled with UHPC, normal-strength concrete and steel fiber-reinforced self-stressing and self-compacting concrete presented a relatively ductile behavior.

The external steel tubes in the middle region were removed by oxygen cutting to obverse the failure mode of the CA-UHPC infill, as presented in Figure 4b. Generally, the concrete infill on the top flange of the specimen was crushed between the two loading points, and many transverse tensile cracks can be observed in the bottom tension region. It is worth noting that the tensile cracks extended to nearly 2/3 of the height of the section for the CA-UHPC core without steel fiber, and the width of cracks was about 2~4 mm. However, for the concrete core with 2% steel fiber, the tensile cracks extended to approximate 1/3 of the height of the beam; both the amount and width of the cracks were decreased considerably compared to the mixture without steel fiber. Furthermore, the crushing of the concrete infill in the compression zone for the CA-UHPC core incorporating 2% steel fiber was weaker than that without steel fiber. With respect to the concrete infill without steel fiber, the compression region was crushed into blocks, whereas only a few tiny cracks were observed for the CA-UHPC with 2% steel fiber.

### 3.2. Overall Deflection Curves

**Figure 5:**
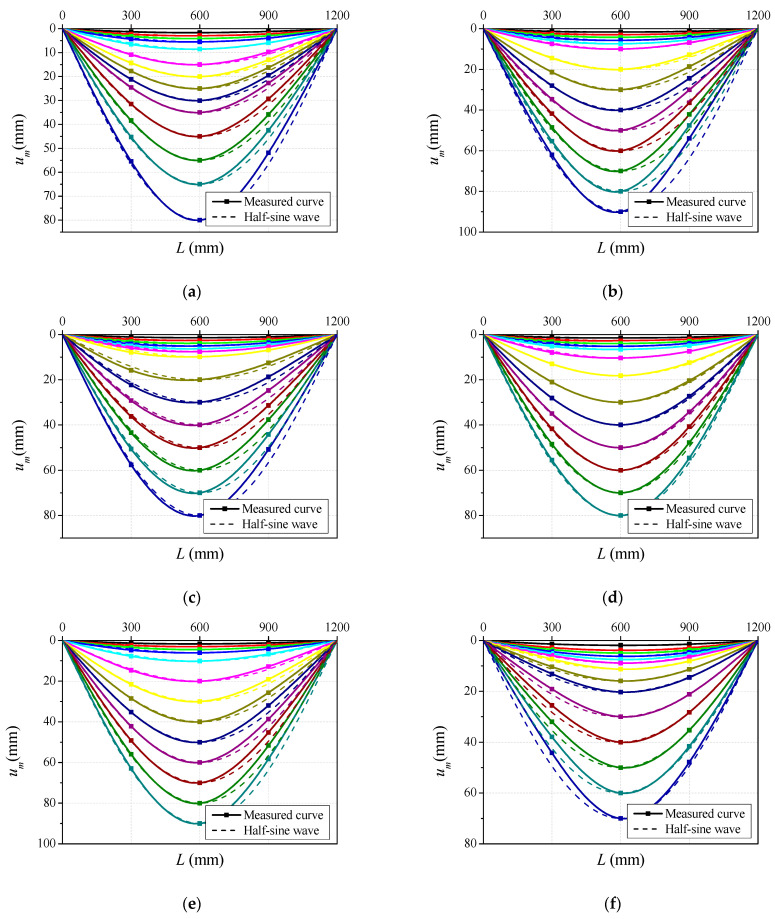
Typical overall lateral deflection curves. (**a**) BC20S6; (**b**) BC30S6; (**c**) BC32S6; (**d**) BC02S6; (**e**) BC32S4.5; (**f**) BC32S8.

The overall deflection curves of the specimens under different load levels are illustrated in Figure 5. The horizontal axis represents the distance to the left support and the vertical axis refers to the lateral deflection, the overall deflection curves at different loading levels are in different colors. Additionally, the half-sine wave curves corresponding to the different deflection levels, marked by the dashed line, are also drawn in the plot. It can be seen from the comparisons that the lateral deflection curves are consistent with the half-sine wave curves at the initial loading stage, with the lateral deflection developing evenly with the applied load, which is similar to the results obtained for normal-strength and high-strength concrete-filled steel tubes under bending load [31,32,33]. Nevertheless, the vertical deflection on both sides of the specimen developed asymmetrically after the specimen entered the plastic stage, and the measured overall deflection curves were inconsistent with the half-sine wave curves. This can be ascribed to the yielding of the steel tube and the asymmetrical cracking of CA-UHPC core in the tensile zone, as well as the crushing of concrete in the compressive zone. Additionally, the steel tube thickness and CA-UHPC type have little impact on the overall deflection curves.

### 3.3. Moment–Curvature Relationship

As shown in Figure 5, the lateral deflection curves of the CA-UHPCFSTs under pure bending load are in agreement with the half-sine curves before the specimen enters the plastic hardening stage; thus, the lateral deflection curves can be expressed by a sinusoidal function:

(1) $$y=-u_{m}\sin(\frac{\pi }{L_{e}}x)$$

where *u*_m_ represents the vertical deflection at the mid-span, *L*_e_ denotes the clear span between the supports, and *x* is the distance to the left support. The sectional curvature (*ϕ*) can be deduced by taking the second derivative:

(2) $$\phi =\frac{\pi^{2}}{L_{e}^{2}}u_{m}\sin\frac{\pi }{L_{e}}x$$

in which *ϕ* represents the sectional curvature. When *x* is set as 0.5*L*_e_, the curvature at the mid-span section (*ϕ*_m_) can be expressed as $\frac{\pi^{2}}{L_{e}^{2}}u_{m}$.

The bending moment (*M*) at the middle one-third span of the beam can be calculated using the following equation:

(3) $$M=\frac{P}{2}\cdot \frac{L_{e}}{3}=\frac{PL_{e}}{6}.$$

**Figure 6:**
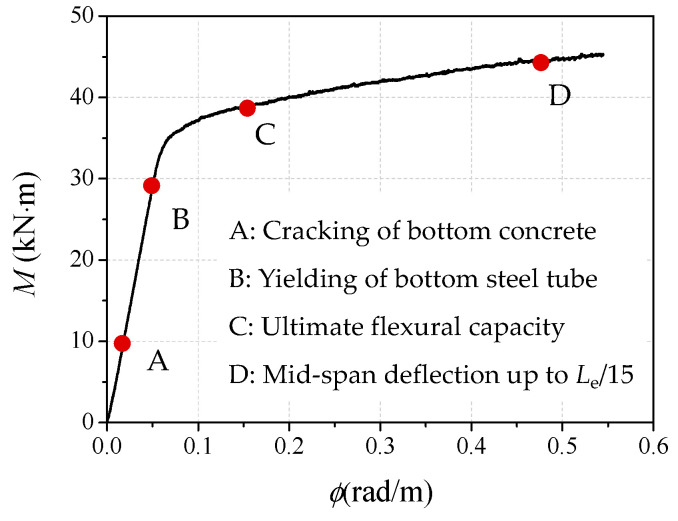
Typical moment (*M*)-versus-curvature (*ϕ*_m_) curve.

The typical moment (*M*)-versus-mid-span curvature (*ϕ*_m_) curve is presented in Figure 6. It is clear that the moment–curvature curves can be divided into three phases: the elastic stage (OB), the elastic–plastic stage (BC) and the plastic hardening stage (CD). Several characteristic points are also labeled in the figure, in which point A represents the initial cracking of the CA-UHPC infill at the tensile zone edge; point B stands for the initial yielding of the steel tube at the tensile flange, corresponding to the proportional limit; point C refers to the tensile strain of the steel tube at the tensile side of the mid-span section, reaching 10,000 *με*; while point D corresponds to the mid-span maximum deflection, up to *L*_e_/15.

(1) Elastic stage: the moment increases linearly with the curvature until the moment reaches 70~80% of ultimate flexural capacity. The moment increases sharply in this stage, while the increase of the curvature is mild. The stiffness remains almost constant, while the steel tube and the compression zone of the CA-UHPC infill remain in an elastic state during this stage. When the load reaches 30~40% of ultimate flexural capacity, the tensile stress in the tensile flange of the concrete exceeds the tensile strength, resulting in transverse cracking. With regard to the core CA-UHPC infilled with steel fiber, the steel fiber is able to restrain the initiation and extension of tensile cracks triggered during this stage, thus enhancing the flexural performance of the concrete core.
(2) Elastic–plastic stage: the moment increases moderately while the curvature increases rapidly. The specimen presents nonlinear response as the gradient of the curves decreases. The yield zone at the bottom flange of the steel tube gradually extends to the compression zone, while the top region of the steel tube and the CA-UHPC are still in an elastic state. The yielding of steel tube tensile flange and the cracking of the concrete core in the tensile zone accelerate the degradation of the section flexural stiffness of the CA-UHPCFST beam. The interaction between the steel tube and the UHPC infill is triggered in this stage, and this can restrict the development of cracks in the CA-UHPC core along the height of the section.
(3) Plastic hardening stage: there is a nearly linear relationship between the bending moment and curvature; however, the slope of the curve is much lower than during the elastic stage. The moment increases at a moderate rate, but nevertheless the curvature increases rapidly. Near point C, the top edge of the steel tube has yielded and the compressive top fiber of CA-UHPC infill is crushed. The stiffness of the specimen decreases dramatically due to the gradual yielding of the steel tube from the tensile flange and the compression edge to the neutral axis. Until the termination of the test, the curve still increases continually, and no distinct softening stage can be observed; the increasing rate of moment in plastic hardening stage is distinctly higher than that of the normal- and high-strength concrete-filled steel tubes or stainless steel tubes. It is demonstrated that the CA-UHPCFST beams under pure bending have better ductility than conventional CFSTs [18,34,35]. This may be ascribed to the higher flexural strength of UHPC and the interaction effect between the steel tube and the CA-UHPC core, where the CA-UHPC prevents the inward local buckling of the steel tube, and accordingly, the further strength degradation and cracking of the core CA-UHPC is restricted due to the confinement provided by the external steel tube.

**Figure 7:**
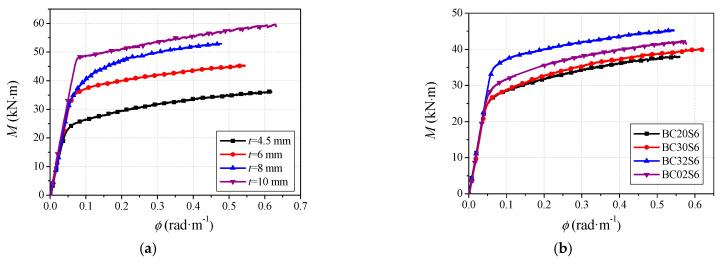
Moment (*M*)-versus-curvature (*ϕ*) curves. (**a**) The effect of steel tube wall thickness; (**b**) the effect of CA-UHPC type.

The moment (*M*)-versus-curvature (*ϕ*) relationships for specimens with different steel tube thicknesses and CA-UHPC strength are plotted in Figure 7. It can be seen from the comparison that specimens with a thicker steel tube thickness have a slightly higher slope of the curve in the elastic stage, and a higher flexural carrying capacity. That is to say, thicker steel tubes have higher flexural resistance and provide a stronger confinement effect to the concrete core. Additionally, a longer elastic stage can be achieved for specimens with thicker steel tubes compared to those with thinner wall thicknesses.

Figure 7b illustrates the effect of CA-UHPC type on the flexural behavior of CA-UHPCFST beams. The specimens of BC20S6 and BC30S6 without steel fibers have similar moment–curvature curves; notably, the hardening strength of BC30S6 is slightly higher than that of BC20S6. This may be attributed to the lower concrete strength of C20; furthermore, it can be verified from Table 3 that the compressive strength of C30 is higher than that of C20. This indicates that, with respect to the CA-UHPC infill without steel fiber, the increased concrete strength has a limited effect on flexural behavior, particularly in the elastic and elastic–plastic stages. Additionally, due to the incorporation of 2% steel fibers in the CA-UHPC infill, the BC32S6 specimen has a longer elastic stage and higher ultimate bending moment. The moment in the plastic hardening stage is also higher than that of BC30S6. Lu et al. [30] reported that the addition of 0.6~1.2% steel fiber can enhance the flexural capacity of steel fiber-reinforced self-stressing and self-compacting concrete-filled steel tube in the range of 1.4~19.9%, since the steel fiber is able to inhibit the initiation of micro-cracks and bridge the cracks, thus remarkably improving the crack resistance, tensile strength and flexural carrying capacity of CA-UHPC infill [6].

With regard to the concrete infilled with steel fiber, the comparison of BC32S6 and BC02S6 demonstrates that the appropriate addition of coarse aggregate in CA-UHPC core results in a longer elastic stage, as well as a higher ultimate flexural capacity and plastic hardening strength. The incorporation of coarse aggregate leads to a denser distribution of steel fiber in the matrix at the same fiber volume fraction, which further enhances the flexural resistance of CA-UHPC infill [6]. However, the slopes of the curves in the elastic and plastic hardening stages seem to be insensitive to the inclusion of steel fiber and coarse aggregate. Overall, the incorporation of steel fiber and a moderate amount of coarse aggregate can significantly improve the flexural bearing capacity, but have a limited effect on flexural stiffness.

### 3.4. Moment–Strain Relationship

**Figure 8:**
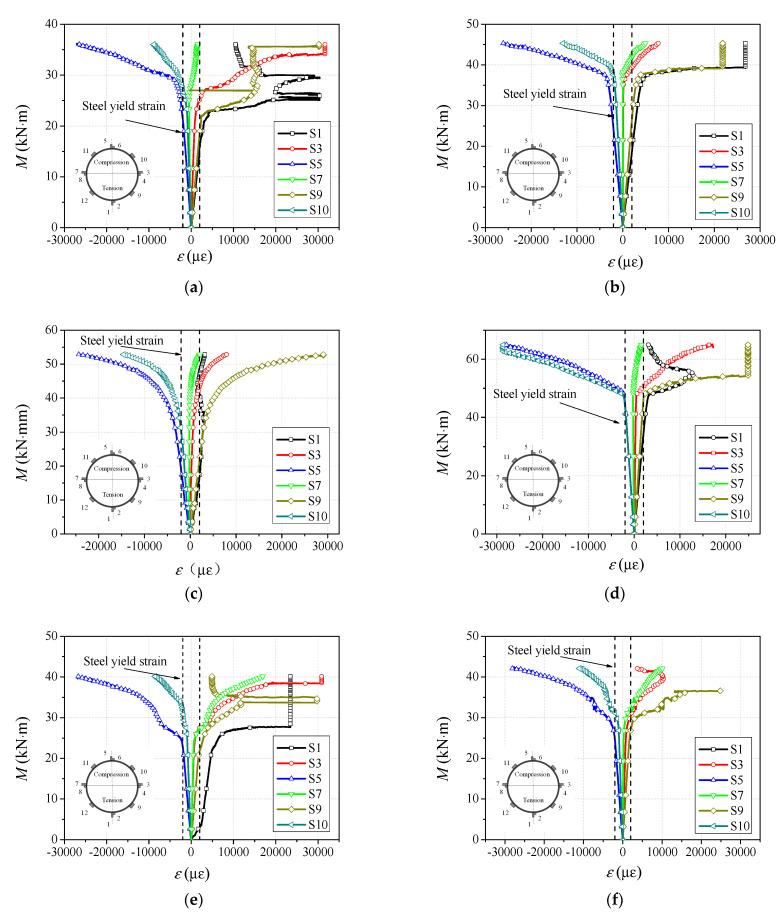
Moment-versus-strain curves of specimens. (**a**) BC32S4.5; (**b**) BC32S6; (**c**) BC32S8; (**d**) BC32S10; (**e**) BC30S6; (**f**) BC02S6.

The typical bending moment (*M*)-versus-extreme fiber compressive and tensile strain (ε) relationships are presented in Figure 8. The yielding strains of steel in the tensile and compressive zones are presented in the figures. It is clear that the moment-versus-strain curves contain an elastic stage, an elastic–plastic stage and a plastic hardening stage. The moment increases linearly with the transverse and longitudinal strains in the elastic stage, with increasing strain in the tensile zone being faster than that in the compressive zone, especially after the bending moment exceeds about 0.4 times the ultimate flexural capacity. When the strain in the middle of the tensile flange exceeds the yield strain of steel, the specimen enters the elastic–plastic stage, followed by the yielding of the steel tube in compressive zone, meaning that the yielding of the steel tube at the tensile flange takes place earlier than that in the compressive zone. When the specimen enters the plastic hardening stage, both the upper and bottom fibers of the steel tube have yielded, and then the strain at mid-span section increases rapidly, while the bending moment increases at a mild rate. The moment–strain curves of specimens with different steel tube thicknesses have similar trends, while the yielding of the thick-walled steel tube occurs earlier than that of the thin-walled steel tube.

**Figure 9:**
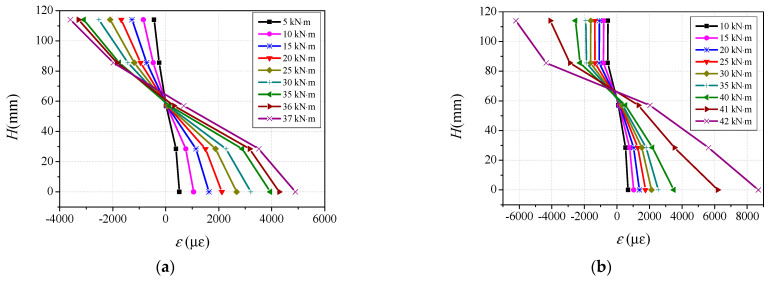
Typical longitudinal strain distribution along the sectional height of the specimen. (**a**) BC32S6; (**b**) BC32S10.

The longitudinal strain distribution along the height of the cross-section at the mid-span can be derived on the basis of the readings of the strain gauges, as shown in Figure 2. The typical longitudinal strain distributions along the height of the cross-section of specimens BC32S6 and BC32S10 under different loading levels are plotted in Figure 9. The horizontal axis represents the measured longitudinal strain, and the vertical axis denotes the height of the measurement point from the bottom flange of the steel tube. It can be observed that the strain distributions along the cross-sectional height remained nearly linear particularly before the plastic stage, the cross-section remains nearly a plane at different loading levels, confirming that the strain distribution along the sectional height meets the plane-section assumption during the loading process. At the initial loading stage, the intersection of the longitudinal strain distribution curves and the strain of ‘0’ is located at the center of the section, meaning that the neutral axis of the specimen coincides with the centroid of the section. When the flexural capacity reaches about 0.4 times the flexural strength, the tensile flange of CA-UHPC infill cracks, and the strain of ‘0’ in the distribution curve gradually moves to the compressive region with increasing flexural moment. Notably, the development in the tensile flange is faster than that at the compression zone edge. After the elastic–plastic stage, the bottom and top flanges of the steel tube yield, and the strain in the tensile zone increases dramatically.

### 3.5. Ultimate Flexural Capacity

**Figure 10:**
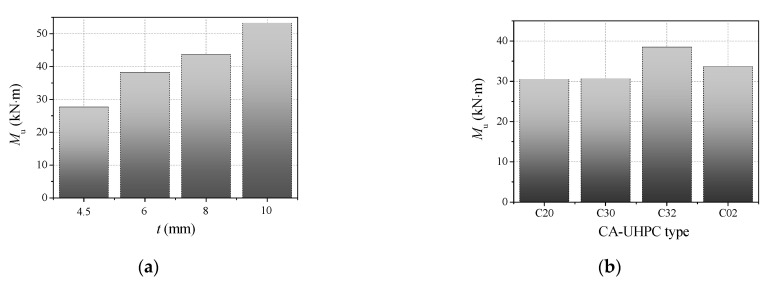
Ultimate flexural capacity of CA-UHPCFST beams. (**a**) The effect of steel tube thickness; (**b**) the effect of CA-UHPC type.

Practically speaking, the bending moment corresponding to the maximum extreme fiber tensile strain of 10,000 *με* is defined as the ultimate flexural capacity (*M*_u_) [34]. Figure 10 presents a comparison of the ultimate flexural capacity of CA-UHPCFSTs with different steel tube thicknesses and CA-UHPC types. The ultimate flexural capacity increases obviously with increasing steel tube thickness. For instance, with an increase in steel tube thickness from 4.5 mm to 10 mm, the ultimate flexural capacity was increased by 38.0%, 57.5% and 92.1%, respectively. This can be ascribed to the increase in steel tube thickness inducing an improvement in composite section strength, while the confinement effect provided by the steel tube on the compression zone of the CA-UHPC infill is also strengthened, thus resulting in a higher compressive strength of the CA-UHPC core at the compressive flange being achieved.

The comparisons presented in Figure 10b indicate that the increase in CA-UHPC strength results in a mild improvement of the ultimate flexural capacity compared to the concrete infill without steel fiber. However, the ultimate flexural capacity was significantly enhanced with the addition of steel fiber. For example, the ultimate flexural capacity increased by 25.6% with the inclusion of 2% steel fiber compared to the values obtained without steel fiber. Compared to BC02S6, with an ultimate flexural capacity of 33.7 kN∙m, the bending moment of BC32S6 increased by 14.3% with the incorporation of 30% granite coarse aggregate by volume fraction. This indicates that the CA-UHPC strength has a mild effect on the ultimate flexural capacity of CA-UHPCFST for CA-UHPC core without steel fiber, which is consistent with the conclusions given by Lu et al. [30] and Han [34], who found that the strength of the concrete core had a limited effect on the ultimate flexural capacity. The inclusion of steel fiber can significantly increase the flexural capacity due to the reinforcement, toughness and cracking resistance of the steel fibers. Furthermore, the addition of moderate coarse aggregate can also strengthen the ultimate flexural capacity at a constant steel fiber dosage. Since the incorporation of coarse aggregate triggers a denser distribution of steel fiber in the matrix, a higher flexural strength of CA-UHPC will be obtained [6]. It can be concluded that the simultaneous addition of moderate coarse aggregate and steel fiber is favorable for enhancing the flexural capacity of CA-UHPCFST beams.

### 3.6. Flexural Stiffness

**Figure 11:**
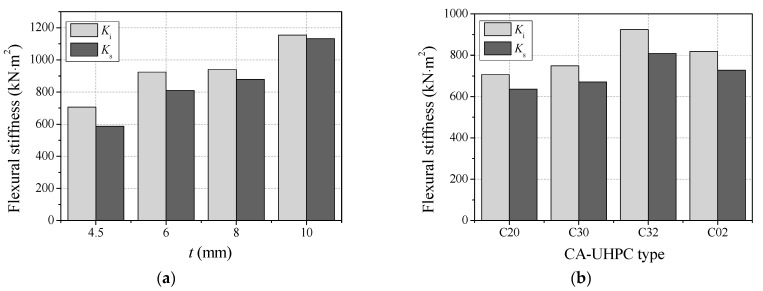
The flexural stiffness of CA-UHPCFSTs. (**a**) The effect of steel tube thickness; (**b**) the effect of CA-UHPC type.

Flexural stiffness is an important factor for determining the elastic deformation of members and evaluating the internal force of structures. The flexural stiffness of concrete-filled steel tubes under in-plane bending can be obtained from the moment–midspan curvature curves. The initial section flexural stiffness (*K*_i_) is defined as the secant stiffness corresponding to the moment of 0.2*M*_u_, while the serviceability-level section flexural stiffness (*K*_s_) represents the secant stiffness corresponding to the moment of 0.6*M*_u_, according to the mechanical model proposed by Han [34]. The initial and serviceability-level flexural stiffness of the tested specimens are summarized in Table 1, and the effects of steel tube thickness and CA-UHPC type on flexural stiffness are plotted in Figure 11. It can be seen from the comparison that the initial flexural stiffness is higher than the serviceability-level section flexural stiffness for the same specimen, indicating that there is an obvious degradation of flexural stiffness of CA-UHPCFSTs in service. Both the initial and serviceability-level flexural stiffness improve significantly with increasing steel tube thickness. This is in agreement with the findings of Li et al. [21], who found that initial flexural stiffness and service-level flexural stiffness increased with an increase in the steel ratio. Furthermore, the initial and serviceability-level flexural stiffness are generally increased with CA-UHPC strength, but this enhancement is not as significant as that obtained with increasing steel tube thickness. The comparison between the CA-UHPC type of C30 and C32 shows that the initial flexural stiffness and the flexural stiffness at the serviceability limit state were also enhanced by the incorporation of 2% steel fiber. Furthermore, the comparison of the C32 and C02 specimens indicates that the incorporation of moderate coarse aggregate in UHPC core also enhances the flexural stiffness of CA-UHPCFST beam, since the coarse aggregate can increase the stiffness and elastic modulus of concrete [36].

### 3.7. Ductility and Energy Dissipation Capacity

The ductility of the structure or the member can be used to assess the inelastic deformation capacity, which the structural member can be subjected to inelastic deformation without losing its load carrying ability prior to failure. The ductility index of the test specimens can be described as follows:

(4) $$\mu =\delta_{u}/\delta_{y}$$

where *δ*_u_ represents the mid-span deflection at the ultimate capacity, and *δ*_y_ denotes the mid-span deflection corresponding to the yield load, which is the applied load corresponding to the yield strain of the steel tube at the tensile flange.

The values of ductility index of CA-UHPCFSTs under bending calculated on the basis of Equation (4) are summarized in Table 5. It can be seen from the comparisons that the ductility index of CA-UHPCFSTs under bending ranged from 2 to 4, with the ratio value of yield moment to the ultimate moment ranging between 0.65 and 0.75. Thus, it can be seen that the yield moment of CA-UHPCFSTs under bending load is approximately 0.7 times the ultimate moment. In addition, the ductility index of members generally decreased with increasing steel tube thickness and CA-UHPC strength, in contrast to the behavior of conventional CFSTs under compression. Compared to the specimen BC32S4.5, with a ductility index of 3.8, the value of *μ* decreased by 16.1%, 24.9% and 40.9% when increasing the steel tube thickness from 4.5 mm to 10 mm, respectively. In comparison to the specimen BC30S6, the ductility index of BC32S6 increased by 13.8% with the incorporation of 2% steel fiber. Moreover, compared to the specimen BC02S2, the ductility index of the specimen BC32S6 increased by 12.0% with the incorporation of 30% coarse aggregate. It can be inferred from these results that the incorporation of steel fiber and moderate coarse aggregate can be expected to improve the ductility of CA-UHPCFSTs.

**Figure 12:**
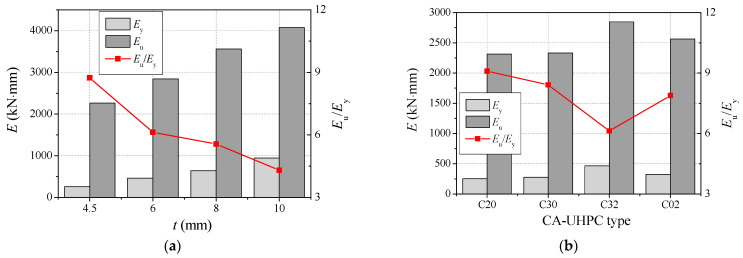
The energy dissipation capacity of CA-UHPCFST members. (**a**) The effect of steel tube thickness; (**b**) the effect of CA-UHPC type.

The energy dissipation capacity is a significant evaluation indicator for structural members, and can be reflected by the area of the load versus the deflection curve. In this study, the energy dissipation index is defined as the quotient between the energy dissipation capacity corresponding to the ultimate capacity and that corresponding to the yield load. The calculated energy dissipation capacity corresponding to the ultimate capacity (*E*_u_) and the yield load (*E*_y_), and the ratio of *E*_u_/*E*_y_ are listed in Table 5 and Figure 12. The energy dissipation capacity increases with increasing steel tube thickness, with an increase in steel tube thickness from 4.5 mm to 10 mm resulting in an improvement in the energy dissipation capacity corresponding to ultimate capacity by 25.6%, 57.3% and 79.8%, respectively. The addition of steel fiber to CA-UHPC improves the energy dissipation capacity, in a manner similar to that of fiber-reinforced concrete-filled steel tubes subjected to bending [30], since the bridging effect of steel fiber can significantly enhance the tensile strength, crack resistance and deformability of concrete. However, the values of *E*_u_/*E*_y_ decrease with the steel tube thickness and CA-UHPC strength, in a manner similar to the ductility index.

### 3.8. Strain Ratio

The strain ratio ($\nu_{\mathrm{sc}}$) is a key parameter for evaluating the confinement effect provided by steel tube on the concrete infill, which is defined as the ratio of the circumferential ratio to the longitudinal strain of steel tube at the cross-section in this paper. The greater the ratio, the stronger the confinement effect on the concrete core. To better understand the mechanical behavior of CA-UHPCFSTs under bending, the composite action between the steel tube and CA-UHPC core is discussed on the basis of an analysis of the strain ratio.

**Figure 13:**
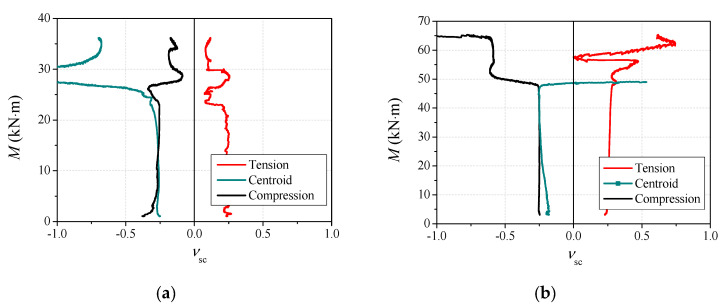
Moment-versus-strain ratio curves. (**a**) *t* = 4.5 mm; (**b**) *t* = 10 mm.

The strain ratios of the tensile, centroidal and compressive flanges at the mid-span section of the members are shown in Figure 13, where the strain ratio in the tensile zone is defined as positive, and in the compression zone it is defined as negative. It can be seen from the comparisons that the strain ratios of the whole section remained almost constant, and were nearly equal to Poisson’s ratio of steel in the elastic stage, indicating that there was almost no confinement effect between the steel tube and the CA-UHPC core, and that the steel tube and the concrete core worked independently. When the specimen entered the elastic–plastic stage, the strain ratios in the compression zone and the centroidal plane increased remarkably, signifying that the steel tube started to provide a confinement effect on the CA-UHPC core. Conversely, the strain ratio in the tensile zone decreased mildly until the termination of the test, indicating that the confinement effect in the tensile zone was small or even negligible. The moment-versus-strain ratio curves in compression zone and centroidal plane changed dramatically after the specimens entered the plastic stage, this can be ascribed to the local buckling of steel tube in these zones. A similar phenomenon was observed in concrete-filled elliptical steel tubes (CFETs) in [37], while the confinement effect in CA-UHPCFST under bending was produced when the applied load reached about 70% of the predicted ultimate load, which is slower than the results obtained for CFET beams, corresponding to 60% of the expected ultimate load. This can be ascribed to the higher flexural stiffness of CA-UHPCFSTs.

## 4. Design Guidelines

Various design codes and specifications were adopted to predict the flexural stiffness and ultimate flexural capacity of circular CA-UHPCFST beams subjected to pure bending load, including EC4 (2004) [19], AISC/LRFD (1999) [24], AIJ (1999) [25], AS 5100.6 (2004) [26], BS 5400-5 (2005) [27], GB 50936 (2014) [28], and the design formula proposed by Han [34].

### 4.1. Comparisons of Flexural Stiffness

The tested initial and serviceability-level flexural stiffness were compared with predictions obtained using the following five design codes or specifications: EC 4, AIJ, AS 5100.6, BS 5400-5, AISC/LRFD. The flexural stiffness of the CFST member under pure bending can be expressed as follows:

(5) $$K_{e}=E_{s}I_{s}+\alpha E_{c}I_{c}$$

where *K*_e_ represents the flexural stiffness, *E*_s_ and *E*_c_ are the elastic modulus of steel and concrete core, respectively, *I*_s_ and *I*_c_ denote the moment of inertia of steel tube and concrete infill, respectively, and *α* is the contribution ratio of concrete core to the section flexural stiffness of the CFST beam.

The flexural stiffness formulas of various codes are listed in Table 6. Furthermore, on the basis of Equation (5), the initial flexural stiffness of circular CA-UHPCFSTs was acquired by fitting the experimental results, as shown in Equation (6).

(6) $$K_{e}=E_{s}I_{s}+0.9E_{c}I_{c}$$

**Figure 14:**
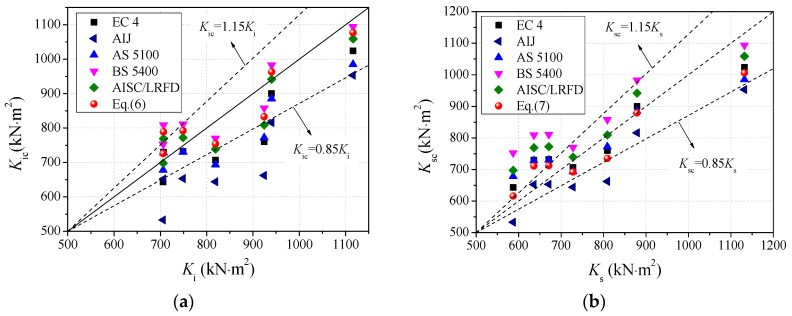
Comparisons of flexural stiffness. (**a**) Initial section flexural stiffness; (**b**) serviceability-level section flexural stiffness.

The initial flexural stiffness obtained in the experimental results is compared with the predictions obtained using various design guidelines and the formula proposed in this paper, as presented in Figure 14a. Table 7 presents the predictions obtained using various design codes and Equation (6), the ratio of the predictions obtained using the codes to the tested value *K*_c_/*K*_i_, and the mean value and standard deviation (coefficient of variation, COV) of this ratio for the different design codes. It is clear that the predictions obtained using EC 4 and AS 5100-6 were conservative, with mean values of 0.926 and 0.925 and COV of 0.077 and 0.078, which are lower than of the values obtained from the experimental results by about 7.4% and 7.5%, respectively. The predictions provided by AIJ were much lower than the test results, since this code mainly considers the contribution of steel tube to the flexural stiffness. BS 5400-6 gave a flexural stiffness about 2.7% higher than the value obtained from testing, and AISC/LRFD gave an initial flexural stiffness about 2.3% lower than the test results. It can be seen from Figure 14a that the predictions provided using Equation (6), proposed in this paper, were in good agreement with the experimental results, making it acceptable for the calculation of the initial flexural stiffness of CA-UHPCFST members.

The tested serviceability-level flexural stiffness (*K*_s_) is compared with the predictions (*K*_sc_) obtained using various design guidelines, as shown in Figure 14b. The predictions, the ratio value of the predictions to the experimental values, the mean value, and the coefficient of variation (COV) of *K*_sc_/*K*_s_ are summarized in Table 8. Additionally, on the basis of the fitting of the current experimental results, a prediction model for the serviceability-level flexural stiffness of CA-UHPCFST beams is proposed as follows:

(7) $$K_{e}=E_{s}I_{s}+0.5E_{c}I_{c}$$

The predictions and the ratios of *K*_sc_/*K*_s_ for Equation (7) are also listed in Table 8. The comparisons in the table clearly indicate that AIJ presents conservative results for the serviceability-level flexural stiffness, with a mean value of 0.912 and a COV of 0.079. The predicted values of serviceability-level flexural stiffness of EC 4 and AS 5100 were slightly higher than of the values obtained from the test results. BS 5400 and AISC/LRFD overestimate the serviceability-level section flexural stiffness of the CA-UHPCFST specimen, with values about 13.8% and 8.2% higher than those obtained during the test, respectively. The prediction obtained using Equation (7) provides the most accurate estimation for the serviceability-level flexural stiffness, with a mean value of 0.997 and a COV of 0.085.

### 4.2. Comparisons of Ultimate Flexural Capacity

The ultimate flexural capacities of circular CA-UHPCFST members under four-point bending load were compared with the predictions made using various design codes and specifications, including EC 4 (1999) [19], AIJ (1997) [25], BS 5400-5 (2005) [27], GB 50936 (2014) [28], and the design method proposed by Han [34]. The current design codes for CFST members are mainly applicable to normal-strength or high-strength concrete-filled steel tubes, and each code has limitations with respect to application scope. The design formulas for the ultimate moment of CFST members under bending are listed in Table 9.

The predicted ultimate flexural capacities (*M*_uc_) of the CA-UHPCFST beams using the various design methods presented in Table 9 were compared with the current experimental results (*M*_u_). The predictions (*M*_uc_), the ratios of the predictions to the tested values (*M*_uc_/*M*_u_), and the mean values and the coefficients of variation (COV) of the ratio *M*_uc_/*M*_u_ are listed in Table 10. It can be seen that AIJ underestimated the flexural capacity of the circular CA-UHPCFST beam, with a mean value of 0.890 and a COV of 0.083. This can be attributed to AIJ only considering the contribution of the steel tube to the flexural capacity, while ignoring the improvement to flexural strength provided by the concrete infill, thus resulting in a lower predicted flexural capacity. Specifically, as mentioned above, the addition of steel fiber improves the flexural capacity of the CA-UHPCFST member. EC 4 overestimated the moment capacity by a range of approximately 8% to 36%, with a mean value of 24.2% and a COV of 0.077. GB 50936 gave a mean value of 1.103 and a COV of 0.226, with the prediction being about 10.3% higher than of the values obtained from the test results; notably, the thicker steel tube led to lower prediction and results with a greater coefficient of variation. The method proposed by Han overestimated the test results, with a mean value of 1.137 and a COV of 0.090. The prediction of BS 5400 was conservative, with a mean value of 0.895 and a COV of 0.073. Since the lowest deviation and coefficient of variation among these design guidelines was obtained using BS 5400-5, we propose that the ultimate flexural capacity of CA-UHPCFST members can be achieved by modifying the design formula of BS 5400-5. Overall, the current design methods for normal-strength and high-strength concrete-filled steel tube beams are inapplicable for calculating the ultimate flexural capacity of CA-UHPCFST beams. Further research needs to be carried out to propose accurate design formulas for the ultimate flexural capacity of CA-UHPCFSTs.

## 5. Conclusions

An experimental investigation was conducted on the flexural behavior of circular CA-UHPC with coarse aggregate-filled steel tubes under a four-point bending load; seven specimens were prepared and tested. The failure modes, moment-versus-curvature curves, moment–strain curves, strain distribution, ductility, flexural stiffness and ultimate flexural capacity were reported. On the basis of the aforementioned analysis, the following conclusions can be drawn:

(1) All the CA-UHPCFST members behaved in a good ductile manner. The failure mode of specimens was similar to that of conventional CFST specimens. The addition of steel fiber had a limited effect on the global failure mode, whereas it was able to effectively reduce the number and depth of the tensile cracks, as well as mitigating the crushing in the compressive zone of the CA-UHPC infill.
(2) The flexural stiffness, ultimate flexural capacity and hardening stage flexural moment were significantly enhanced with increased steel tube thickness. The increase in concrete strength for CA-UHPC without steel fiber had a limited effect on the flexural capacity. The incorporation of steel fiber was able to moderately increase the ultimate flexural capacity of the CA-UHPCFST members as well as lengthen the elastic stage of moment-versus-curvature curve.
(3) The yield flexural moment of the CA-UHPCFST members under bending was approximately 0.7 times the ultimate flexural strength. The confinement effect in the compressive zone and the centroidal plane was triggered after the specimen entered the elastic–plastic stage, while the confinement effect in the tensile flange was minor or even negligible throughout the whole loading process.
(4) Two empirical formulas were developed to predict the initial flexural stiffness and serviceability-level flexural stiffness of circular CA-UHPCFSTs. EC 4, AIJ, AS 5100 presented conservative results for the initial flexural stiffness, BS 5400-5 and AISC/LRFD gave the most accurate prediction on the value of *K*_i_. Additionally, EC 4, AS 5100, BS 5400-5 and AISC/LRFD overestimated the serviceability-level flexural stiffness.
(5) The current design rules are imprecise for calculating the ultimate flexural moment of CA-UHPCFSTs, with AIJ and BS 5400 presenting conservative results for the flexural capacity of circular CA-UHPCFST beams subjected to pure bending load, while EC 4, GB 50936, and the formula proposed by Han overestimate the ultimate flexural capacity. Further research is still required to propose accurate design formulas for the ultimate flexural capacity of CA-UHPCFSTs.

## Figures and Tables

**Table 1:** Details of the specimens.

Specimen	*L* (mm)	*D* (mm)	*t* (mm)	*f*_y_ (MPa)	*A*_s_ (mm^2^)	*f*_ck_ (MPa)	*A*_c_ (mm^2^)	*ξ*	*M*_u_ (kN∙m)	*K*_e_ (kN∙m^2^)	
										*K*_i_	*K*_s_
BC20S6	1400	114	6	406	2035	101	8167	1.00	30.52	709	636
BC30S6	1400	114	6	406	2035	108	8167	0.94	30.66	749	671
BC32S6	1400	114	6	406	2035	124	8167	0.82	38.52	924	809
BC02S6	1400	114	6	406	2035	112	8167	0.90	33.68	819	728
BC32S4.5	1400	114	4.5	420	1547	124	8655	0.61	27.72	706	587
BC32S8	1400	114	8	400	2662	124	7539	1.14	43.66	940	879
BC32S10	1400	114	10	429	3285	124	6936	1.63	53.24	1154	1132

Note: *L* stands for the length of specimen, *D* and *t* represent the external diameter and nominal wall thickness of steel tube, *f*_y_ and *A*_s_ denote the yield strength and sectional area of steel tube, and *f*_ck_ and *A*_c_ stand for the 28-day prismatic compressive strength and cross-sectional area of the CA-UHPC core. *ξ* represents the confinement index, $\xi =f_{y}A_{s}/f_{c}A_{c}$; greater values of *ξ* indicate a stronger confinement effect provided by the steel tube on the concrete core. *M*_u_ refers to the ultimate flexural capacity, and *K*_i_ and *K*_s_ represent the initial and serviceability-level flexural stiffness, respectively.

**Table 2:** Material properties of steel tubes.

Thickness (mm)	*f*_y_ (MPa)	*f*_u_ (MPa)	*E*_s_ (GPa)
4.5	420	603	205
6	406	589	206
8	400	592	206
10	429	587	205

**Table 3:** Mix proportions and mechanical properties of CA-UHPC.

Mix	Content (kg/m^3^)									*f*_cu_ (MPa)	*f*_ck_ (MPa)	*E*_c_ (GPa)
	Cement	Silica Fume	Fly Ash	Steel Fiber	PPF	Water	SP	Sand	Granite			
C20	599.24	138.29	184.38	0.0	0.9	165.94	23.05	1014.10	430	113	101	39
C30	493.49	113.88	151.84	0.0	0.9	136.66	18.98	835.14	817	117	108	42
C32	449.22	103.67	138.22	157.0	0.9	124.40	17.28	760.22	817	139	124	48
C02	660.72	152.47	203.30	157.0	0.9	182.97	25.41	1118.13	0	126	112	41

Note: *f*_cu_ and *f*_ck_ represent the cubic and prismatic compressive strength, respectively, *E*_c_ represents the elastic modulus of the CA-UHPC infill.

**Table 4:** Chemical compositions of cementitious materials.

Composition	Na_2_O	MgO	Al_2_O_3_	SiO_2_	SO_3_	Fe_2_O_3_	P_2_O_5_	CaO	K_2_O	MnO	ZnO	SrO
Cement	0.079	2.14	4.5	19.58	3.06	3.119	0.128	64.94	0.75	0.127	0.024	0.148
Silica fume	0.068	0.224	0.354	92.87	1.26	0.113	0.11	0.213	0.332	0.008	0.019	0.005
Fly ash	0.552	0.575	30.63	48.74	0.706	2.611	0.247	2.44	1.25	0.016	0.013	0.060

**Table 5:** Ductility and energy dissipation indexes of CA-UHPCFSTs under bending.

Specimen	*M*_y_ (kN∙m)	*M*_u_ (kN∙m)	*δ*_y_ (mm)	*δ*_u_ (mm)	*E*_y_ (kN∙m)	*E*_u_ (kN∙m)	*M*_y_/*M*_u_	*μ* = *δ*_u_/*δ*_y_	*E*_u_/*E*_y_
BC20S6	20.04	30.52	5.14	20.22	254.42	2313.45	0.66	3.93	9.09
BC30S6	20.8	30.66	5.63	20.43	277.05	2330.89	0.68	3.63	8.41
BC32S6	28.96	38.52	6.28	20.04	464.46	2845.67	0.75	3.19	6.13
BC02S6	22.63	33.68	5.92	21.16	325.15	2562.51	0.67	3.57	7.88
BC32S4.5	18.91	27.72	5.63	21.41	259.34	2264.70	0.68	3.80	8.73
BC32S8	32.72	43.66	8.34	23.78	640.45	3561.25	0.75	2.85	5.56
BC32S10	38.52	53.24	10.56	23.70	944.75	4072.25	0.72	2.24	4.31

**Table 6:** Flexural stiffness formulas of the design codes for CFSTs.

Design Codes	Formula
EC 4 (2005) [19]	$K_{e}=E_{s}I_{s}+0.6E_{c}I_{c}$
AISC/LRFD (1999) [24]	$K_{e}=E_{s}I_{s}+0.8E_{c}I_{c}$
AS 5100.6 (2004) [26]	$K_{e}=0.9E_{s}I_{s}+0.9E_{c}I_{c}$
AIJ (1997) [25]	$K_{e}=E_{s}I_{s}+0.2E_{c}I_{c}$
BS 5400-5 (2005) [27], GB 50936 (2014) [28]	$K_{e}=E_{s}I_{s}+E_{c}I_{c}$

**Table 7:** Comparisons of the measured initial flexural stiffness with the predictions of the design guidelines.

Specimen Label	*K*_i_ (kN∙m^2^)	EC 4		AIJ		AS 5100		BS 5400		AISC/LRFD		Equation (6)	
		*K*_ic_	*K*_ic_/*K*_i_	*K*_ic_	*K*_ic_/*K*_i_	*K*_ic_	*K*_ic_/*K*_i_	*K*_ic_	*K*_ic_/*K*_i_	*K*_ic_	*K*_ic_/*K*_i_	*K*_ic_	*K*_ic_/*K*_i_
BC20S6	707	730	1.03	652	0.92	728	1.03	809	1.14	769	1.09	789	1.12
BC30S6	749	732	0.98	653	0.87	730	0.98	811	1.08	772	1.03	792	1.06
BC23S6	924	760	0.82	662	0.72	772	0.84	858	0.93	809	0.87	833	0.90
BC02S6	819	707	0.86	644	0.79	693	0.85	770	0.94	739	0.90	754	0.92
BC23S4.5	706	643	0.91	533	0.76	678	0.96	753	1.07	698	0.99	726	1.03
BC23S8	940	900	0.96	816	0.87	885	0.94	983	1.05	942	1.00	963	1.02
BC23S10	1116	1024	0.92	953	0.85	985	0.88	1094	0.98	1059	0.95	1077	0.97
Mean value		0.926		0.825		0.925		1.027		0.977		1.002	
COV		0.077		0.090		0.078		0.078		0.076		0.077	

**Table 8:** Comparisons of the measured serviceability-level flexural stiffness with the predictions of design guidelines.

Specimen Label	*K*_s_ (kN∙m^2^)	EC 4		AIJ		AS 5100		BS 5400		AISC/LRFD		Equation (7)	
		*K*_sc_	*K*_sc_/*K*_s_	*K*_sc_	*K*_sc_/*K*_s_	*K*_sc_	*K*_sc_/*K*_s_	*K*_sc_	*K*_sc_/*K*_s_	*K*_sc_	*K*_sc_/*K*_s_	*K*_sc_	*K*_sc_/*K*_s_
BC20S6	636	730	1.15	652	1.02	728	1.14	809	1.27	769	1.21	711	1.12
BC30S6	671	732	1.09	653	0.97	730	1.09	811	1.21	772	1.15	712	1.06
BC23S6	809	760	0.94	662	0.82	772	0.95	858	1.06	809	1.00	735	0.91
BC02S6	728	707	0.97	644	0.89	693	0.95	770	1.06	739	1.02	692	0.95
BC23S4.5	587	643	1.10	533	0.91	678	1.15	753	1.28	698	1.19	616	1.05
BC23S8	879	900	1.02	816	0.93	885	1.01	983	1.12	942	1.07	879	1.00
BC23S10	1132	1024	0.90	953	0.84	985	0.87	1094	0.97	1059	0.94	1006	0.89
Mean value		1.025		0.912		1.024		1.138		1.082		0.997	
COV		0.088		0.079		0.105		0.105		0.096		0.085	

**Table 9:** Design formulas for the ultimate strength of CFST under bending.

Codes	Formulation
EC4 [19]	$M_{u}=f_{y}[A_{s}(D-2t-d_{c})/2+Dt(t+d_{c})]$ $,\;d_{c}=\left(A_{s}-2dt\right)/[(d-2t)\rho +4t]$ $,d=0.6f_{\mathrm{ck}}/f_{y}$
AIJ [25]	$M_{u}=Zf_{y}$ $,Z=(D^{3}-{\left(D-2t\right)}^{3})/6$
BS 5400-5 [27]	$M_{u}=0.91Sf_{y}(1+0.01m)$ $,S=t^{3}{\left(D_{e}/t-1\right)}^{2}$
GB 50936 [28]	$M_{u}=\gamma_{m}W_{\mathrm{sc}}f_{\mathrm{sc}}$ $,\;f_{\mathrm{sc}}=\left(1.212+B\theta +C\theta^{2}\right)f_{c}$ $,\;W_{\mathrm{sc}}=\pi \left(r_{0}^{4}-r_{\mathrm{ci}}^{4}\right)/4r_{0}$ $,\;\theta =A_{s}f_{y}/A_{c}f_{c}$
Han [34]	$M_{u}=\gamma_{m}\cdot W_{\mathrm{scm}}\cdot f_{\mathrm{scy}}$ $,f_{\mathrm{scy}}=\left(1.14+1.02\xi \right)\cdot f_{\mathrm{ck}}$ $,\;\gamma_{m}=1.1+0.48\cdot \ln(\xi +0.1)$ $,\;\xi =A_{s}f_{y}/A_{c}f_{c}$

**Table 10:** Comparisons of the predictions and the test results for ultimate flexural capacity.

Specimen Label	*M*_u_ (kN∙m)	EC 4		AIJ		BS 5400-5		GB 50936		Han	
		*M*_uc_	*M*_uc_/*M*_u_	*M*_uc_	*M*_uc_/*M*_u_	*M*_uc_	*M*_uc_/*M*_u_	*M*_uc_	*M*_uc_/*M*_u_	*M*_uc_	*M*_uc_/*M*_u_
BC20S6	30.5	41.5	1.36	29.5	0.97	29.5	0.97	38.6	1.27	36.4	1.19
BC30S6	30.6	41.5	1.35	29.5	0.96	29.7	0.97	39.1	1.28	36.6	1.19
BC32S6	38.5	41.7	1.08	29.5	0.77	30.1	0.78	41.3	1.07	37.6	0.98
BC02S6	33.7	41.6	1.23	29.5	0.88	29.9	0.89	39.9	1.19	36.9	1.10
BC32S4.5	27.7	33.5	1.21	23.2	0.84	23.9	0.86	36.8	1.33	29.6	1.07
BC32S8	43.6	52.3	1.20	38.1	0.87	38.2	0.87	42.7	0.98	49.9	1.14
BC32S10	53.2	67.1	1.26	50.1	0.94	48.9	0.92	32.6	0.61	68.8	1.29
Mean value		1.242		0.890		0.895		1.103		1.137	
COV		0.077		0.083		0.073		0.226		0.090	

## Data Availability

Data available on request due to restrictions eg privacy or ethical.
